# Risk factors for leptospirosis and brucellosis in people living with human immunodeficiency virus who attended a referral hospital in southeastern Brazil

**DOI:** 10.1590/0037-8682-0076-2021

**Published:** 2021-07-02

**Authors:** Flavio Gonçalves Brito, Benedito Donizete Menozzi, Karine Bott Mantovan, Alexandre Naime Barbosa, Cassiano Victória, Helio Langoni, Rodrigo Costa da Silva

**Affiliations:** 1 Universidade Estadual Paulista “Júlio de Mesquita Filho”, Campus Botucatu, Faculdade de Medicina, Programa de Doenças Tropicais, Botucatu, SP, Brasil.; 2 Universidade Estadual Paulista “Júlio de Mesquita Filho”, Campus Botucatu, Faculdade de Medicina Veterinária e Zootecnia, Departamento de Produção Animal e Medicina Veterinária Preventiva, Botucatu, SP, Brasil.; 3 Universidade do Oeste Paulista (Unoeste), Faculdade de Ciências Agrárias, Presidente Prudente, SP, Brasil.

**Keywords:** Anthropozoonosis, People living with HIV/AIDS, Public health

## Abstract

**INTRODUCTION:**

Leptospirosis and brucellosis cause immunosuppression that worsens the clinical condition of people living with HIV/AIDS (PLWHA). We investigated the serological profile and risk factors of PLWHA.

**METHODS:**

Serum samples (n=238) were researched for *Brucella* spp. antibodies using Rose Bengal and tube agglutination tests and *Leptospira* spp. antibodies using the microscopic agglutination test.

**RESULTS:**

All samples were negative for *Brucella* spp. For leptospirosis, four samples (1.69%) were positive, and Andamana was the prevalent serovar.

**CONCLUSIONS:**

Low or no detection of these zoonoses does not reduce their importance in PLWHA. Vigilant, educational, and preventive measures should be adopted.

The emergence and re-emergence of certain zoonoses have increased in recent years, requiring knowledge and updating health professionals. Some of these diseases are characterized by opportunistic conditions, such as coinfection with immunosuppressive diseases, such as acquired immunodeficiency syndrome (AIDS)^1^ ^,^ ^2^.

Since the onset of the Brazilian epidemic scenario from 1980 to June 2019, 966,058 cases of AIDS have been accounted for in the country, 633,462 (65.6%) in men and 332,505 (34.4%) in women. According to the data provided by the Ministry of Health, it is the largest number that was reported in Southeastern Brazil (495,587; 51.3%)^1^.

Brucellosis is an anthropozoonosis that affects a range of different species of animals, including humans, and is caused by *Brucella* spp., a small, gram-negative coccobacillus. It is transmitted by direct contact with an infected animal, indirectly by contact with contaminated secretions and excretions^2^, or by ingesting contaminated food, particularly milk and milk derivatives produced with unboiled or unpasteurized milk^3^. *Brucella* may occur either as smooth (S) or rough (R) species. These two types are based on the aspect of colonies on agar plates, which is in accordance with the cell surface and lipopolysaccharide (LPS) structure^4^. There are 10 recognized *Brucella* species. Among them, the S-LPS species are *Brucella melitensis* (*B. melitensis*), *Brucella suis* (*B. suis*), and *Brucella abortus* (*B. abortus*), whereas the R-LPS species are *Brucella ovis* (*B. ovis*) and *Brucella canis* (*B. canis*). All these species are clinically and epidemiologically important to animal and human health. Although underdiagnosed, > 500,000 new human cases occur annually, mainly in developing countries^2^ ^,^ ^5^.

On the contrary, leptospirosis is worldwide distributed, with the majority of cases and diseases occurring in tropical and subtropical regions, and in developing countries^6^. Several mammalian species are infected by *Leptospira,* but only a few act as efficient reservoirs capable of establishing long-term kidney colonization and shedding bacteria in the urine^7^. It occurs mainly in rats, the universal carriers of leptospirosis, and production animals, such as cattle and sheep. Close contact with animals increases the risk of human infection. In an urban scenario, dogs are the main source of infection for humans and are also considered sentinels and carriers for the disease^6^.

Considering the importance of brucellosis and leptospirosis as anthropozoonoses, particularly in immunosuppressed patients, the purpose of this study was to determine *Brucella* spp. and *Leptospira* spp. antibodies and related risk factors in people living with HIV/AIDS (PLWHA) in a specialized infectious disease outpatient clinic in a referral hospital in southeastern Brazil.

This was a cross-sectional study and patients treated at the Domingos Alves Meira Specialized Infectious Diseases Outpatient Service (SAEI-DAM) of the Clinical Hospital (HC) of Botucatu Medical School, São Paulo State University (FMB-UNESP) were sampled. Botucatu is located in the mid-west region of São Paulo State (22º53’09” S; 48º26’42” W) with an estimated population of 146,497^8^.

The SAEI-DAM registered patients were accompanied by a multidisciplinary team. The medical record system of the HC-FMB-UNESP was used to access patient data. Among them, 300 PLWHA in several towns in the study area were identified, but only 238 patients met the study requirements: 129 (54.2%) men and 109 (45.8%) women, aged 18-76 years. No pregnant women were identified during the study period. Each patient was included in the study after obtaining an informed consent form.

Blood samples were collected using a vacutainer without anticoagulant by cephalic vein puncture to detect specific antibodies against each disease. Blood samples were centrifuged at 1,600 × *g* for 10 min, and the serum samples were stored at -4°C. In addition, an epidemiological questionnaire (“social and demographic characteristics”, “water, garbage, and sewer variables”, and “host-related characteristics”) was applied to the PLWHA to determine the risk factors related to the studied disease.

The present study was approved by the Research Ethics Committee of the FMB-UNESP (protocol #821261).

*B. abortus* and *B. suis* antibodies were researched using the Rose Bengal test (RBT), a serum agglutination test in buffered acid-antigen stained with Rose Bengal, and the slow tube agglutination test with 2-mercaptoethanol (SAT-2ME) and without 2ME (SAT)^5^.

*Leptospira* spp. antibodies were researched using the microscopic agglutination test (MAT)^9^. Cultures of *Leptospira* spp. standard serovars, maintained by weekly subcultures in Ellinghausen-McCullough-Johnson-Harris liquid medium, were used as antigens. Twenty-eight serovars were used: Australis, Bratislava, Autumnalis, Butembo, Castellonis, Bataviae, Canicola, Whitcombi, Cynopteri, Djasiman, Sentot, Grippotyphosa, Hebdomadis, Copenhageni, Icterohaemorraghiae, Javanica, Panama, Pomona, Pyrogenes, Hardjo-Prajitno, Hardjo-Miniswajezak, Hardjo-C.T.G., Hardjo-Bovis, Wolffi, Shermani, Tarassovi, Andamana, and Patoc. Serum samples were considered reagents for the presence of agglutination (≥ 50%) after challenge to the serovars, considering a cut-off titer of 100.

Descriptive statistics were used to determine the absolute and relative frequencies of positive samples for one or both zoonoses, and analytical statistics were used to determine any associations with epidemiological variables. Therefore, the results of serological tests were analyzed in association with the epidemiological variables by univariate analysis using the Chi-square test (χ^2^) and/or Fisher’s exact test. Subsequently, all variables that presented p-value ≤ 0.05, in the univariate analysis, were included in the multivariate analysis and the logistic regression model^10^. All analyses were performed using Epi Info^TM^ software, v.7.2.0.1, with a significance level (?) of 5%.

All samples were negative for antibodies against *B. abortus* and *B. suis*.

*Leptospira* spp. antibodies were detected in 4/238 (1.68%; 95% confidence interval [CI] 0.68-4.23%) serum samples, which was lower than that observed in Tanzania (9/203; 4.43%)^11^. The reagent PLWHAs comprehended 3/129 (2.32%; 95%CI 0.84-6.60) male and 1/109 (0.9%; 95%CI) female, 100% were 30-60 years old, 75% completed high school, but not college, and 100% earned up to five minimum wages (Table 1). The results concerning water resources and waste and sewage management are presented in Table 2, whereas those concerning the hosts are presented in Table 3. Only 1/4 (25%) samples reacted to Pyrogenes serovar (titer 200) and 3/4 (75%) to Andamana (titers 200, 400, and 800). Regarding the epidemiological variables, only the “occurrence of floods when it rained” presented a significant association (p-value = 0.00), with 4/33 (12.12%) reagent patients who experienced this important risk factor.

**TABLE 1:** Association (univariate analysis) between the *Leptospira* spp. antibody research and the social and demographic variables regarding the studied population

Variable	N	n	% (95%CI)^a^	OR^b^	p-value^c^
Sex					
Male	129	3	2.3 (0.8-6.6)	0.4 (0.0-3.8)	0.63^d^
Female	109	1	0.9 (0.2-5.0)		
Age					
15 < x ≤ 30 years	28	0	0.0 (0.0-11.9)	-	0.87^d^
30 < x ≤ 45 years	94	3	3.2 (1.2-9.0)		
45 < x ≤ 60 years	89	1	1.1 (0.3-6.0)		
60 < x ≤ 76 years	17	0	0.0 (0.0-18.5)		
uninformed	10	0	0.0 (0.0-28.5)		
Marital status					
Married	92	2	2.2 (0.7-7.6)	-	0.42^d^
Single	92	1	1.1 (0.3-5.9)		
Be living together	10	1	10.0 (2.3-41.3)		
Divorced	29	0	0.0 (0.0-11.6)		
Widowed	14	0	0.0 (0.0-21.8)		
Educational level					
Undergraduate	24	0	0.0 (0.0-13.7)	-	0.63^d^
Incomplete undergraduation	17	1	5.9 (1.4-27.3)		
Completed the high school	67	2	3.0 (0.9-10.2)		
Incomplete high school	21	0	0.0 (0.0-15.4)		
Completed the primary/secondary school	39	0	0.0 (0.0-8.8)		
Incomplete primary/secondary school	61	1	1.6 (0.4-8.7)		
No educational level	4	0	0.0 (0.0-52.2)		
Monthly wage					
up to 2 minimum wage	165	3	1.8 (0.7-5.2)	-	1.00^d^
3-5 minimum wage	58	1	1.7 (0.4-9.1)		
6-10 minimum wage	9	0	0.0 (0.0-30.8)		
>10 minimum wage	4	0	0.0 (0.0-52.2)		
Residence					
Urban area	198	3	1.5 (0.6-4.3)	1.8 (0.2-17.4)	0,51^d^
Rural area	38	1	2.6 (0.6-13.5)		
Have you heard about leptospirosis or brucellosis?					
No	78	1	1.3 (0.3-6.8)	1.0 (0.1-11.4)	1.00^d^
Yes	154	2	1.3 (0.4-4.6)		

**Legend: N:** total number of sampled patients; **n:** number of positive patients for the microscopic agglutination test (MAT); ^a^ **%:** percentage (95%CI, 95% confidence interval); ^b^ **OR:** *Odds Ratio*; ^c^ p-value for a = 5%; ^d^ Fisher’s exact test.

**TABLE 2:** Association (univariate analysis) between the *Leptospira* spp. antibody research and the water, garbage, and sewer variables.

Variable	N	n	% (95%CI)^a^	OR^b^	p-value^c^
Do you drink tap water?					
No	85	1	1.2 (0.3-6.3)	1.7 (0.2-16.5)	1.00^e^
Yes	152	3	2.0 (0.7-5.6)		
Water source					
Filtered water					
Yes	91	0	0.0 (0.0-3.9)	-	0.30^e^
No	146	4	2.8 (1.1-6.8)		
Tap water					
No	105	1	1.0 (0.2-5.1)	2.4 (0.2-23.6)	0.63^e^
Yes	132	3	2.3 (0.8-6.4)		
Spout’s water					
No	237	4	1.7 (0.7-4.2)	-	1.00^e^
Yes	0	0	0.0 (0.0-0.0)		
Artesian well water					
No	223	3	1.4 (0.5-3.9)	5.6 (0.5-58.1)	0.22^e^
Yes	14	1	7.1 (1.7-32.0)		
Mineral water					
No	214	4	1.9 (0.8-4.7)	-	1.00^e^
Yes	23	0	0.0 (0.0-14.2)		
Does you have water tank?					
No	46	2	4.4 (1.3-14.5)	0.2 (0.0-1.7)	0.18^e^
Yes	187	2	1.1 (0.3-3.8)		
How often is the water tank cleaned?					
Semiannual	23	0	0.0 (0.0-14.2)	-	0.24^e^
Monthly	1	0	0.0 (0.0-84.2)		
Annual	80	0	0.0 (0.0-4.4)		
Biannual	26	1	3.8 (0.9-19.0)		
Never	58	1	1.7 (0.4-9.1)		
Sewer destination					
Public sewer system	207	3	1.4 (0.5-4.2)	-	0.40^e^
Septic tank	25	1	4.0 (1.0-19.6)		
Open sky	3	0	0.0 (0.0-60.2)		
Rivers / streams	0	0	0.0 (0.0-0.0)		
When it rains, does it flood the street?					
No	203	0	0.0 (0.0-1.8)	-	0.00^e^
Yes	33	4	12.1 (5.0-27.4)		
What is the destination of your home garbage?					
Public collect	228	4	1.8 (0.7-4.4)	-	1.00^d^
Wasteland	0	0	0.0 (0.0-0.0)		
Backyard	0	0	0.0 (0.0-0.0)		
Burning trash	8	0	0.0 (0.0-33.6)		

**Legend: N:** total number of sampled patients; **n:** number of positive patients for the microscopic agglutination test (MAT); ^a^ **%:** percentage (95%CI, 95% confidence interval); ^b^ **OR:** *Odds Ratio*; ^c^ p-value for a = 5%; ^d^ Chi-square test; ^e^ Fisher’s exact test.

**TABLE 3:** Association (univariate analysis) between the *Leptospira* spp. antibody research and the epidemiological variables related to the hosts.

Variable	N	n	% (95%CI)^a^	OR^b^	p-value^c^
Do you have animal at home?					
No	51	1	2.0 (0.5-10.3)	0.8 (0.1-8.1)	1.00^d^
Yes	185	3	1.6 (0.6-4.6)		
Which species?					
Dog					
No	29	1	3.4 (0.8-17.2)	0.4 (0.0-4.1)	0.40^d^
Yes	158	2	1.3 (0.4-4.5)		
Cat					
No	131	2	1.5 (0.5-5.4)	1.2 (0.1-13.2)	1.00^d^
Yes	56	1	1.8 (0.4-9.4)		
Bird					
No	146	2	1.4 (0.4-4.8)	1.8 (0.2-20.4)	0.53^d^
Yes	41	1	2.4 (0.6-12.6)		
Pig					
No	178	3	1.7 (0.6-4.8)	-	1.00^d^
Yes	8	0	0.0 (0.0-33.6)		
Wild animal					
No	180	3	1.7 (0.6-4.8)	-	1.00^d^
Yes	7	0	0.0 (0.0-36.9)		
What is the food source to the animal(s)?					
Animal food (kibble)					
No	11	1	9.1 (2.1-38.5)	0.1 (0.0-1.4)	0.17^d^
Yes	169	2	1.2 (0.4-4.2)		
Homemade food					
No	128	2	1.6 (0.5-5.5)	1.3 (0.1-14.2)	1.00^d^
Yes	51	1	2.0 (0.5-10.3)		
Leftovers					
No	179	3	1.7 (0.6-4.8)	-	1.00^d^
Yes	0	0	0.0 (0.0-0.0)		
Raw meat					
No	172	3	1.7 (0.6-5.0)	-	1.00^d^
Yes	7	0	0.0 (0.0-36.9)		
Where does the animal stay?					
Home (all day)	123	1	0.8 (0.2-4.4)	-	0.06^d^
Street (all day)	5	1	20.0 (4.3-64.1)		
Home + Street	47	1	2.1 (0.5-11.1)		
If at home, where does the animal stay?					
Inside home	68	2	2.9 (0.9-10.1)	-	0.65^d^
Backyard	97	1	1.0 (0.2-5.6)		
Inside home + backyard	11	0	0.0 (0.0-26.5)		
Have you already found rats at home?					
No	96	0	0.0 (0.0-3.7)	-	0.14^d^
Yes	130	4	3.1 (1.2-7.6)		

**Legend: N:** total number of sampled patients; **n:** number of positive patients for the microscopic agglutination test (MAT); ^a^ **%:** percentage (95%CI, 95% confidence interval); ^b^ **OR:** *Odds Ratio*; ^c^ p-value for a = 5%; ^d^ Fisher’s exact test.

Brucellosis and leptospirosis are very important to veterinary science and public health because of their severity and lethality in humans^2^. Brucellosis is not a mandatory notifiable disease in Brazil for humans and may be underdiagnosed. In addition, no organized public health network exists in Brazil to identify human cases^5^. Globally, the prevalence of brucellosis in PLWHA ranges from 5.98% to 73.33% in Iran^2^ ^,^ ^3^ ^,^ ^12^ to 66.67% in Spain^13^. This range may be related to regional cultural habits (namely, raw milk ingestion), exposure to infected animals, and/or positive family history of brucellosis^3^, and reinforces the importance of periodic serological surveys to improve disease monitoring and surveillance, especially in PLWHA. Even with negative results for the detection of *B. abortus* and *B. suis* antibodies in PLWHA in this study, its prevalence in PLWHA from developing countries may be five times higher, according to the World Health Organization (WHO)^4^.

Both humoral and cellular immune responses are required for brucellosis because the elimination of bacteria occurs in the intracellular environment. This fact increases the susceptibility of HIV/AIDS patients to *Brucella* infection^12^. Brucellosis is rare in PLWHA, although the eradication of intracellular bacteria is largely dependent on cellular immunity. In this way, it is hypothesized that HIV infection does not increase the incidence of brucellosis because most cases occur in asymptomatic patients with preserved immunity, and the epidemiology, clinical presentation, diagnosis, response to the therapy, and outcomes are similar to those observed in HIV-negative patients. A cross-sectional study carried out in basic health units from Alagoas State, Brazil, reported 4.4% *Brucella* spp. antibodies in patients with brucellosis; however, no notification of the disease was identified in the Notifiable Diseases Information System^5^.

The close contact between humans and animals is evident and may indicate a related risk factor^6^. The role of rodents in the transmission of many diseases, including leptospirosis, is widely known^7^. In urban areas, rodents are important reservoirs and sources of *Leptospira* infection with a higher probability of infection during rainy periods, mainly in tropical areas of developing and undeveloped countries^6^.

Although certain risk factors may be considered as indicators of the dissemination or, even, the severity of the disease in PLWHA, namely, tap water or artesian well water as “water source”, “if it floods when it rains” (Table 2), and even “if the animal stays at home or in the street” (Table 3), the low prevalence and sampled population limit the adequate characterization of the possible and eligible risk factors. The association between each variable and the serology results suggests a possible risk for PLWHA that experienced floods after rain. Despite this limitation, a higher seroprevalence was observed in males from urban areas, which could be related to the occupational risk. In Pernambuco State, Brazil, the authors also reported a higher occurrence of infection in male patients^14^. In non-PLWHA, leptospirosis has a high impact as an occupational disease. This fact was observed in São Paulo State, Brazil, among blood donors (1.3% reagents) from the Donor Center of the Clinical Hospital, FMB-UNESP^9^. This finding reinforces the relevance of continuous epidemiological surveillance and health education actions to control the disease in both animals and humans.

The observed range of the serological results seems reasonable, considering the different geographic regions and variations in environmental conditions, including rainfall, temperature and humidity, serovars, quality of the antigens, and interpretation of the results. Based on the serological results, the present study confirms that *Leptospira* spp. were circulating in the PLWHA population from São Paulo State, probably maintained by the animal population, even with low prevalence.

The low or no detection of the studied zoonoses does not reduce their importance in causing disease in PLWHA. Therefore, vigilant, educational, and preventive measures should be developed and maintained for the early identification of factors that predispose to the occurrence of these zoonoses.
